# Urban-Adjacent Soils as a Global Reservoir of Toxigenic and Antimicrobial-Resistant *Bacillus cereus* Sensu Lato

**DOI:** 10.3390/ijms27188293

**Published:** 2026-09-17

**Authors:** Marek Bartoszewicz, Milena Maliszewska, Urszula Czyżewska

**Affiliations:** 1 Department of Microbiology and Biotechnology, Faculty of Biology, University of Bialystok, Ciolkowskiego 1J Street, 15-245 Bialystok, Poland; 2 Biotechnology Students Scientific Club, Faculty of Biology, University of Bialystok, Ciolkowskiego 1J Street, 15-245 Bialystok, Poland; milena.maliszewska04@gmail.com; 3 Laboratory of Cytobiochemistry, Faculty of Biology, University of Bialystok, Ciolkowskiego 1J Street, 15-245 Bialystok, Poland; urszula.czyzewska@uwb.edu.pl

**Keywords:** *Bacillus cereus* sensu lato, antimicrobial resistance, urban soils, phylogeography, multilocus sequence typing, enterotoxins, minimum inhibitory concentration, environmental reservoir

## Abstract

*Bacillus cereus* sensu lato (*B. cereus* s.l.) is ubiquitous in soil, but the extent to which urban-adjacent soils act as reservoirs of antimicrobial-resistant and potentially toxigenic lineages remains poorly understood globally. We analysed 243 *B. cereus* s.l. isolates representing unique sequence types from ten urban locations worldwide. Population structure was assessed by multilocus sequence typing and phylogenetic analyses, antimicrobial susceptibility by disk diffusion and MIC determination, and the presence and expression of *nheA*, *hblA*, and *cytK* by PCR and qPCR. Isolates from the same locations clustered on the phylogenetic tree more frequently than expected by chance, although isolates from different regions remained widely intermingled. Selected lineages also showed significant latitudinal distributions. Antimicrobial susceptibility varied markedly among locations, with frequent ampicillin resistance and geographic differences in cumulative elevated MIC burden that were not consistently associated with local phylogenetic clustering. These patterns may reflect region-specific anthropogenic selective pressures. The *nheA* gene was detected in all isolates, whereas *hblA* and *cytK* occurred in approximately half, and neither gene carriage nor relative expression was associated with elevated MIC burden. Urban-adjacent soils therefore harbour phylogenetically diverse *B. cereus* s.l. populations with widespread enterotoxin-associated genetic potential and geographically heterogeneous antimicrobial resistance.

## 1. Introduction

*Bacillus cereus* sensu lato (*B. cereus* s.l.) comprises a group of closely related spore-forming bacteria that are ubiquitous in both natural environments and anthropogenically influenced ecosystems [1]. Members of this group have been isolated from a broad range of ecological niches, including soil, freshwater and marine environments, food matrices, plants, and both invertebrate and vertebrate hosts [1]. Among these habitats, soil is considered the primary ecological reservoir of *B. cereus* s.l., providing conditions conducive to long-term persistence and genetic diversification [1]. Despite this fundamental environmental role, *B. cereus* s.l. has traditionally been investigated mainly in the context of food safety, which has led to a narrowed perception of this bacterial group as a problem primarily associated with food contamination and generally mild, sporadic cases of foodborne illness [2].

In addition to the widespread occurrence of typically commensal environmental isolates, *B. cereus* s.l. also encompasses opportunistic pathogens capable of causing human disease. The pathogenic potential of these bacteria is largely linked to the biosynthesis of a diverse repertoire of toxins, including the non-haemolytic enterotoxin (NHE), haemolysin BL (HBL), cytotoxin K (CytK), cereulide, EntFM, and other virulence-associated factors [3]. These toxins are responsible for two well-characterised clinical forms of food poisoning: the diarrhoeal and the emetic syndromes [3,4]. However, *B. cereus* s.l. may also act as an etiological agent of extraintestinal infections, including wound infections, bacteraemia, endophthalmitis, and respiratory tract infections, particularly in immunocompromised individuals [5]. Owing to substantial genotypic and phenotypic similarity among members of the group, as well as frequent horizontal gene transfer (HGT), the classification of *B. cereus* s.l. isolates at the species level remains challenging. Consequently, ecotype-based approaches are increasingly applied, or isolates are reported at the collective level of *B. cereus* s.l. [6]. In contrast, *Bacillus anthracis* is notably more distinct from the remaining members of the group and is associated with the most severe clinical outcomes. Although phylogenetically embedded within *B. cereus* s.l., it represents a relatively discrete genetic and epidemiological lineage.

Interestingly, selected soil- and plant-associated *B. cereus* strains have also been investigated as plant growth-promoting bacteria and biological control agents because of their ability to promote nutrient acquisition, produce growth-stimulating compounds, induce systemic resistance, and suppress phytopathogens. However, the potential agricultural use of *B. cereus s.l.* requires careful biosafety assessment, as isolates displaying desirable plant-associated traits may also harbour enterotoxin or other virulence determinants. Consequently, evaluation of toxigenic potential and antimicrobial resistance should accompany the selection of *B. cereus* strains intended for agricultural bioinoculant applications [7,8].

Compared with other clinically relevant bacteria, *B. cereus* s.l. remains relatively underrepresented in studies addressing antimicrobial resistance (AMR), particularly in the context of environmental reservoirs [2]. This gap persists despite a growing body of evidence reporting resistant phenotypes among isolates recovered from food, including products derived from organic and minimally processed production systems [9,10]. Resistance to β-lactams, macrolides, aminoglycosides, and other antimicrobial classes has been described, suggesting that *B. cereus* s.l. may constitute a relevant environmental reservoir of resistance determinants and potentially contribute to their further dissemination [10]. In food production systems, particularly those of animal origin, selective pressures favouring the development of antimicrobial resistance are most commonly associated with the use of antibiotics in animal husbandry and agriculture [11,12,13,14].

Similarly, soils located in proximity to large urban agglomerations represent environments exposed to complex anthropogenic pressures, including the presence of pharmaceutical residues, heavy metals, biocides, and microorganisms originating from municipal waste streams [15,16,17,18]. Heavy metals and biocides may promote the co-selection of antibiotic resistance genes through shared genetic carriers and common regulatory mechanisms [17,19]. Such conditions can favour the selection and persistence of antimicrobial resistance even in bacterial populations not directly exposed to antibiotic therapy [16,20].

To date, it remains unclear whether soils located in the vicinity of large urban agglomerations constitute a globally relevant reservoir of *B. cereus* s.l. lineages combining toxigenic potential with antimicrobial resistance, or whether these traits are restricted to particular phylogenetic groups, geographical regions, or locally exposed populations [1,2]. The present study therefore aimed to determine whether urban-adjacent soil isolates of *B. cereus* s.l. recovered from geographically distant locations represent a shared pool of potentially hazardous bacteria and to establish how antimicrobial resistance phenotypes and toxin-associated genetic determinants are related to their phylogenetic affiliation and geographical origin. By integrating population structure, antimicrobial susceptibility profiles, and toxin gene repertoires, we sought to test whether clinically relevant traits are globally distributed across environmental *B. cereus* s.l. populations or are shaped predominantly by lineage-specific and regional factors.

## 2. Results

### 2.1. B. cereus s.l. Strain Origin and Genetic Population Structure

**Figure 1:**
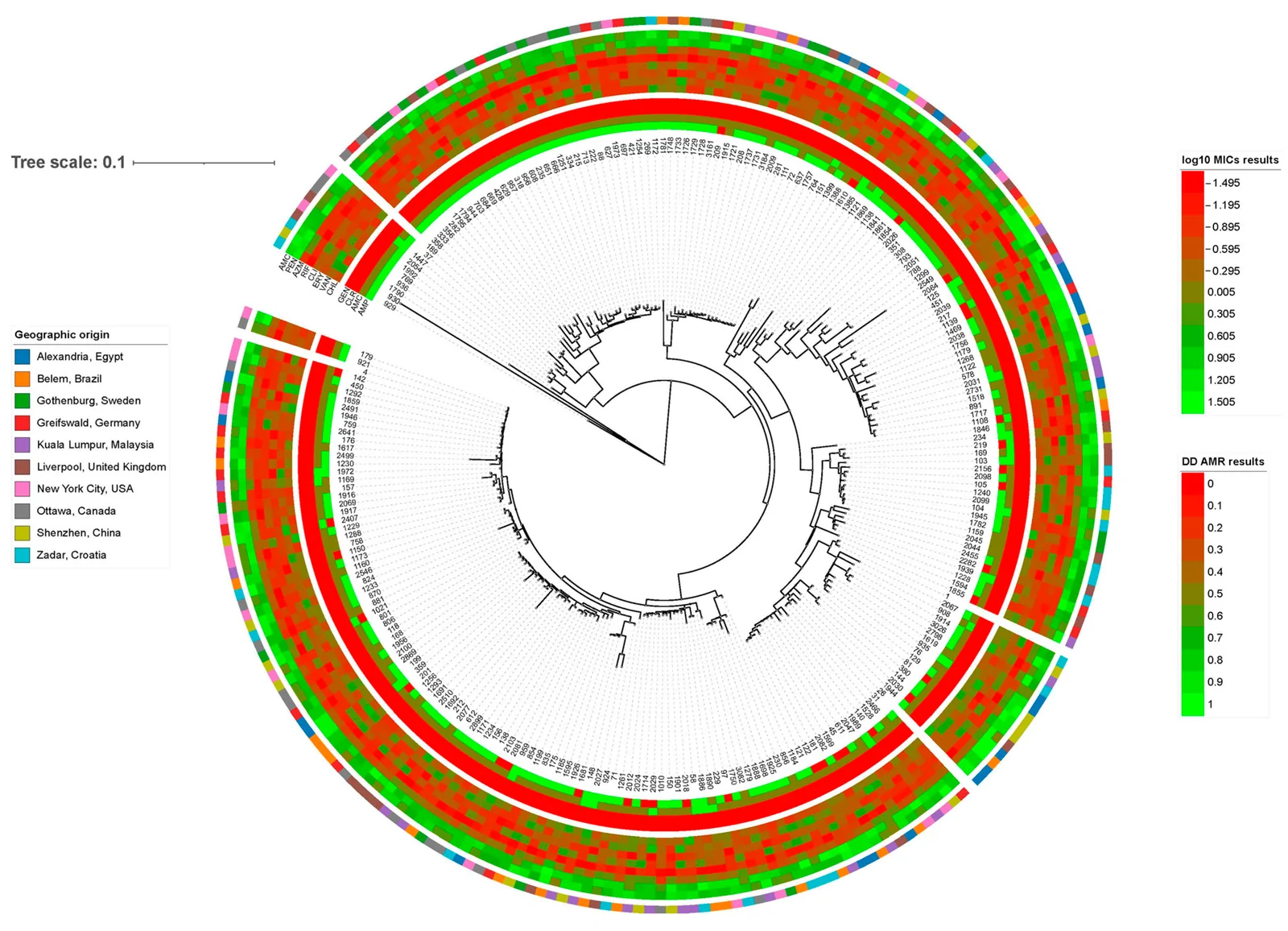
Phylogenetic population structure of *B. cereus* s.l. in relation to geographical origin and antimicrobial resistance. The dendrogram (Maximum Likelihood, GTR+G+I) includes 243 *B. cereus* s.l. strains and four reference strains (*B. anthracis* Ames, *B. cereus* ATCC 14579, *B. cereus* F4810/72, and *B. mycoides* DSM 11821). Phylogeny was reconstructed using MEGA 6, and phylogenetic, resistance profile, and geographic origin data were integrated and visualised using iTOL. The outer ring indicates the geographical origin of the isolates, whereas the inner rings represent disk diffusion results and log_10_-transformed MIC values, encoded using a colour gradient. Full resolution dendrograms (traditional and circular) is accessible in Supplementary Files (Supplementary S1).

The analysed dataset comprised 243 *B. cereus* s.l. strains. In accordance with the study design, isolates from each location represented unique sequence types (STs), enabling the assessment of phylogenetic and phylogeographic patterns independently of local clonal dominance. Phylogenetic reconstruction revealed a heterogeneous population structure characterised by clearly delineated evolutionary lineages (Figure 1). Although isolates from different geographical regions were largely dispersed across the tree topology, certain clades showed enrichment for strains originating from specific locations. Notably, within the lineage related to the reference psychrotolerant strain *B. mycoides* DSM 11821, (German Collection of Microorganisms and Cell Cultures) isolates from higher latitudes were more frequently represented.

Permutation-based tests of phylogeographic association demonstrated a significant geographical structure within the population. Both the Association Index (AI = 729) and the Parsimony Score (PS = 158) were significantly lower than expected under random distribution of geographical origin across the phylogeny (AI random = 883.9 ± 21.1; PS random = 177.9 ± 3.8; permutation test, *p* = 0.0005 for both statistics). These results indicate non-random clustering of isolates originating from the same geographical locations within the phylogenetic tree.

### 2.2. Local Phylogenetic Structure

The strongest location-specific signals of monophyletic clustering were observed for Belém (Brazil), Liverpool (United Kingdom), and Shenzhen (China), each of which contained a monophyletic cluster comprising three local isolates (MC = 3; nominal permutation *p* = 0.0290, 0.0385, and 0.0285, respectively). However, none of these location-specific associations remained statistically significant after correction for multiple testing using the Benjamini–Hochberg false discovery rate procedure (adjusted *p* = 0.1283 for all three locations, Table 1).

For the remaining locations, observed MC values ranged from 0 to 2 and were not significant even before multiple-testing correction. Thus, although the global AI and PS statistics demonstrated significant phylogeographic structure, evidence for strong location-specific monophyletic clustering was comparatively weak and heterogeneous across sampling sites.

Given that the samples originated from geographically distinct locations characterised by different climatic conditions, it was justified to compare latitude data with the genetic relatedness of the isolates. An overrepresentation of isolates from low- and mid-latitude regions (primarily Alexandria, Shenzhen, and Kuala Lumpur) was observed within the clade that also grouped isolates related to *B. anthracis* (ST-1) and the emetic *B. cereus* strain F4810/72 (ST-26).

In contrast, the lineage encompassing the mesophilic reference strain *B. cereus* ATCC 14579 included 23 study isolates; among them, seven originate from New York (approximately 40.7° N) and Greifswald (approximately 54.1° N). The mean latitude of isolates clustered within this lineage was approximately 39° N. Isolates phylogenetically closer to the psychrotolerant strain *B. mycoides* DSM 11821 (ST-189) were characterised by a higher mean latitude of approximately 51°N. This clade was predominantly composed of isolates from Gothenburg (57.7° N), Greifswald (54.1° N), Liverpool (53.4° N), and Ottawa (45.4° N).

In summary, permutation testing (10,000 iterations) indicated that the virulent clade (including the reference strains *B. anthracis* Ames and *B. cereus* F4810/72) exhibited a significantly lower median latitude compared with the remainder of the population (mean 25.4° N vs. 39.7° N; permutation test, *p* = 0.003). The mesophilic clade containing *B. cereus* ATCC 14579 showed no significant latitudinal shift (*p* = 0.42), whereas the psychrotolerant clade represented by DSM 11821 was significantly shifted northwards (mean 50.8° N vs. 33.1° N; *p* = 0.001).

### 2.3. Antimicrobial Resistance

**Figure 2:**
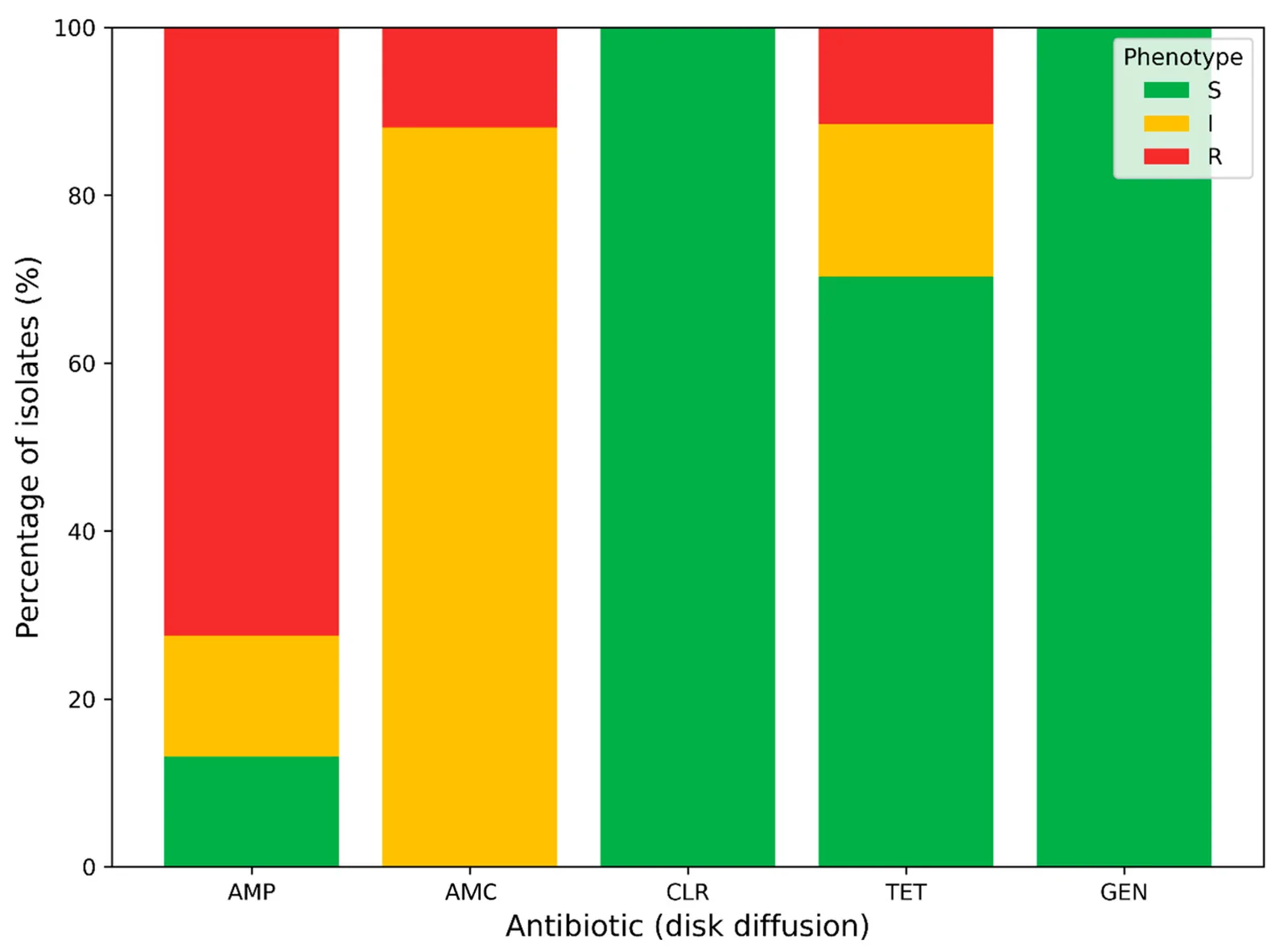
Antimicrobial susceptibility profiles of *B. cereus* s.l. isolates determined by disk diffusion assays. The stacked 100% bar chart presents the proportion of isolates classified as susceptible (S), intermediate (I), or resistant (R) according to CLSI-based interpretative criteria for each antibiotic evaluated using the disk diffusion method. AMP—ampicillin; AMC—amoxicillin/clavulanic acid; CLR—clarithromycin; TET—tetracycline; GEN—gentamicin.

Antimicrobial susceptibility was assessed using the disk diffusion (DD) method, with selected antibiotics additionally evaluated using gradient strip tests. Disk diffusion assays revealed pronounced differences in susceptibility profiles depending on the antibiotic tested (Figure 2). The highest proportion of resistant strains was observed for ampicillin (72.4%), whereas for amoxicillin/clavulanic acid, isolates with intermediate susceptibility predominated (88.1%). All isolates were susceptible to clarithromycin and gentamicin. In contrast, tetracycline displayed a mixed distribution of phenotypes, with a predominance of susceptible strains (70.4%).

Chi-square analysis demonstrated a significant association between geographical origin and the frequency of the resistant phenotype for both ampicillin (χ^2^ = 57.0; *p* < 0.001) and tetracycline (χ^2^ = 22.2; *p* = 0.014). Higher frequencies of resistance were observed, among others, in Alexandria, Shenzhen, and Kuala Lumpur, whereas susceptible phenotypes predominated in Gothenburg, Greifswald, and Ottawa.

**Figure 3:**
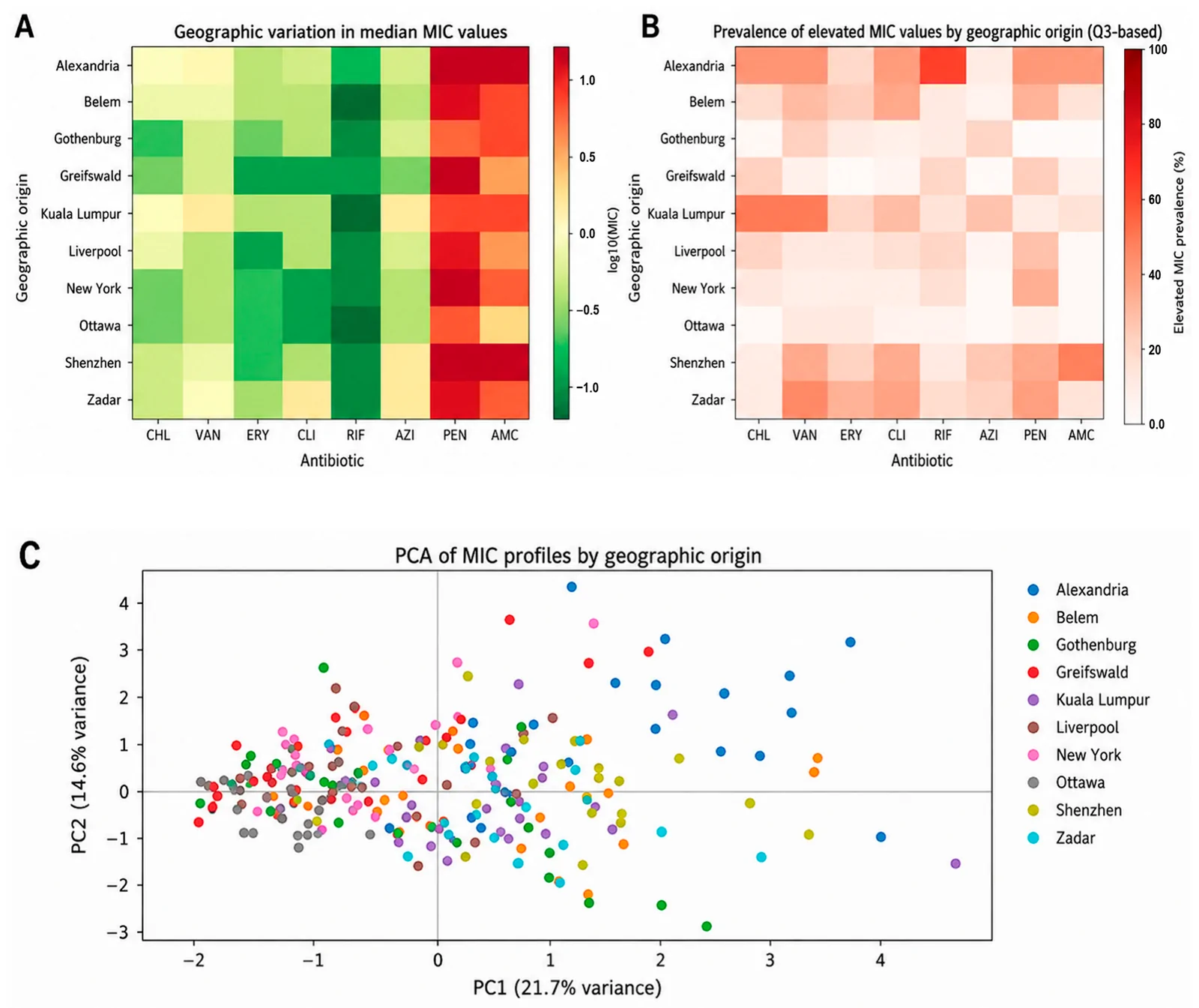
Geographic variation in minimum inhibitory concentration (MIC) profiles among *B. cereus* sensu lato isolates. (**A**) Heatmap showing median MIC values for individual antibiotics across geographic locations. Values are presented as log_10_-transformed MICs, with the colour scale ranging from lower (green) to higher (red) median values. (**B**) Geographic distribution of elevated MIC values. For each antibiotic, an elevated MIC was defined as a value exceeding the third quartile (Q3) of the overall MIC distribution; colour intensity represents the percentage of isolates from each location exceeding this threshold. (**C**) Principal Component Analysis (PCA) of MIC profiles. Each point represents an individual isolate, with colours indicating geographic origin. PC1 and PC2 explain 21.7% and 14.6% of the total variance, respectively (36.3% combined). Reference strains were excluded from these analyses. CHL, chloramphenicol; VAN, vancomycin; ERY, erythromycin; CLI, clindamycin; RIF, rifampicin; AZI, azithromycin; PEN, penicillin; AMC, amoxicillin/clavulanic acid.

Variation in MIC distributions across locations. Minimum inhibitory concentration (MIC) data enabled quantitative assessment of antimicrobial susceptibility among isolates. Median MIC values differed between geographical locations for several of the tested compounds (Figure 3A), revealing regional shifts in susceptibility profiles. To facilitate comparisons between therapeutics active at different concentration ranges, calculated medians were presented on a log_10_ scale.

Uniformly elevated median MIC values for multiple antibiotics were observed in Alexandria, Shenzhen, and Kuala Lumpur. In contrast, Gothenburg, Greifswald, and Ottawa were characterised by lower and more homogeneous MIC distributions, while the remaining locations displayed intermediate profiles.

Relative to global medians, Alexandria exhibited elevated median MIC values for 7 of 8 antibiotics tested. Shenzhen, Kuala Lumpur, Belém, and Zadar showed elevated medians for 4 of 8 antibiotics, whereas Gothenburg and Ottawa did not display elevated medians for any of the tested compounds.

The prevalence of isolates with MIC values exceeding the antibiotic-specific Q3 thresholds varied substantially across locations and antibiotics (Figure 3B). Alexandria exhibited the highest overall prevalence of elevated MIC values across the analysed compounds. In contrast, Gothenburg and Ottawa showed generally low prevalences of elevated MIC values, although small proportions of isolates exceeded the Q3 threshold for selected antibiotics. The remaining locations displayed intermediate and antibiotic-dependent profiles.

Principal Component Analysis (PCA) based on MIC values further highlighted differences in resistance profiles among isolates. The first two principal components (PC1—representing a global gradient of elevated MIC values; PC2—reflecting contrasts in resistance profiles) accounted for a substantial proportion of the total variance and revealed partial clustering of isolates according to geographical origin (Figure 3C).

Although considerable overlap between regions was observed, certain locations occupied distinct positions within the PCA ordination space, indicating divergent multidimensional MIC profiles driven by specific combinations of antimicrobial susceptibility values.

### 2.4. Isolate-Level Burden of Elevated MICs

**Figure 4:**
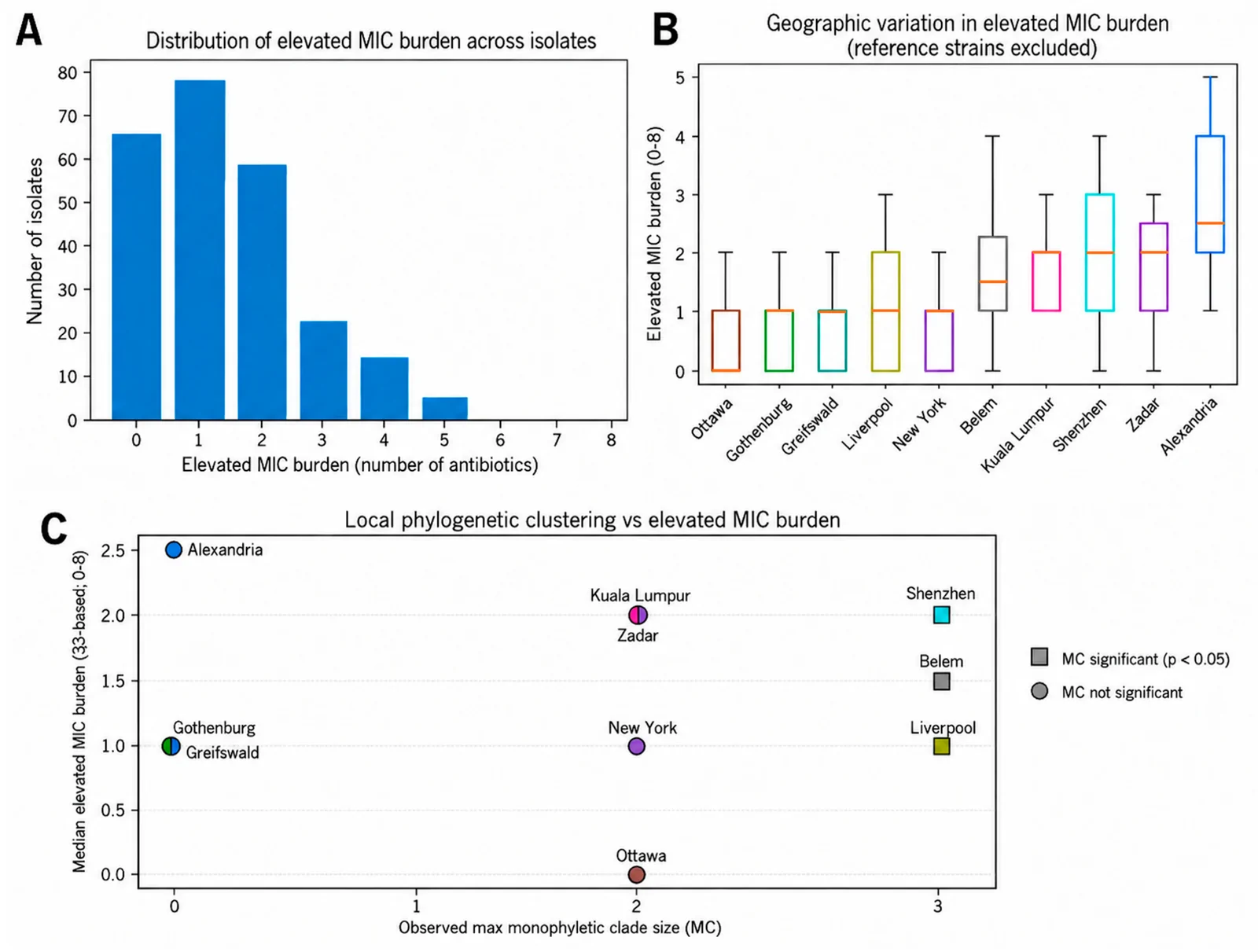
Distribution and geographic variation in cumulative elevated MIC burden and its relationship with local phylogenetic clustering in *B. cereus* sensu lato isolates. (**A**) Distribution of cumulative elevated MIC burden across the study population. For each isolate, the burden was defined as the number of antibiotics (range 0–8) for which the MIC exceeded the third quartile (Q3) of the overall distribution for that antibiotic. (**B**) Geographic variation in cumulative elevated MIC burden. Boxplots show the distribution of burden values among isolates from individual sampling locations; boxes represent the interquartile range (Q1–Q3), horizontal lines indicate medians, and whiskers extend to the most extreme values within 1.5 × IQR. Colours correspond to geographic origin. (**C**) Relationship between local phylogenetic clustering and median cumulative elevated MIC burden at the sampling-location level. The *x*-axis represents the maximum observed size of a monophyletic clade composed of isolates from the same location (MC), whereas the *y*-axis shows the median elevated MIC burden for that location. Squares indicate locations showing nominal evidence of local monophyletic clustering (permutation *p* < 0.05), whereas circles indicate locations with nominal *p* ≥ 0.05. None of the location-specific MC associations remained statistically significant after Benjamini–Hochberg false discovery rate correction.

The cumulative burden of elevated MIC values (MIC burden) was defined as the number of antibiotics (range 0–8) for which a given isolate exhibited MIC values exceeding the third quartile (Q3) of the global distribution. The distribution of this metric across the entire population was broad (Figure 4A). While a substantial proportion of isolates displayed a low burden (0–1 elevated MIC values), isolates exhibiting concurrent elevation of MICs for multiple antibiotics were also observed.

Cumulative MIC burden differed between geographical locations (Figure 4B). Alexandria was characterised by the highest median burden, with the distribution shifted towards higher levels of cumulative resistance. In contrast, Gothenburg and Ottawa exhibited low median burdens, with a predominance of isolates showing 0–1 elevated MIC values. The remaining locations, including Shenzhen, Kuala Lumpur, Belém, and Zadar, displayed intermediate distributions, with a moderate proportion of isolates exhibiting multiple elevated MICs.

### 2.5. Integration of Phylogenetic Structure and Antimicrobial Resistance

To explore the relationship between local phylogenetic structure and antimicrobial resistance, cumulative MIC burden was compared with the degree of monophyletic clustering at the level of geographical locations.

Locations showing nominal evidence of local monophyletic clustering, such as Shenzhen and Belém, exhibited moderately elevated median MIC burdens (Figure 4C). However, high MIC burden values were also observed in locations where no evidence of local phylogenetic clustering was detected, most notably in Alexandria. Conversely, several locations characterised by phylogenetically intermixed population structures displayed low levels of cumulative resistance burden.

Collectively, the analyses indicate that the *B. cereus* s.l. population exhibits significant, yet heterogeneous, geographical structuring at the phylogenetic level. Antimicrobial resistance profiles, assessed both by disk diffusion and MIC-based approaches, differ across regions and reveal complex patterns that cannot be explained solely by clonal expansion of local lineages. Elevated MIC burden occurs both within locally clustered phylogenetic lineages and among isolates representing diverse phylogenetic backgrounds.

### 2.6. Distribution and Expression of Enterotoxin Genes Across the B. cereus s.l. Population

**Figure 5:**
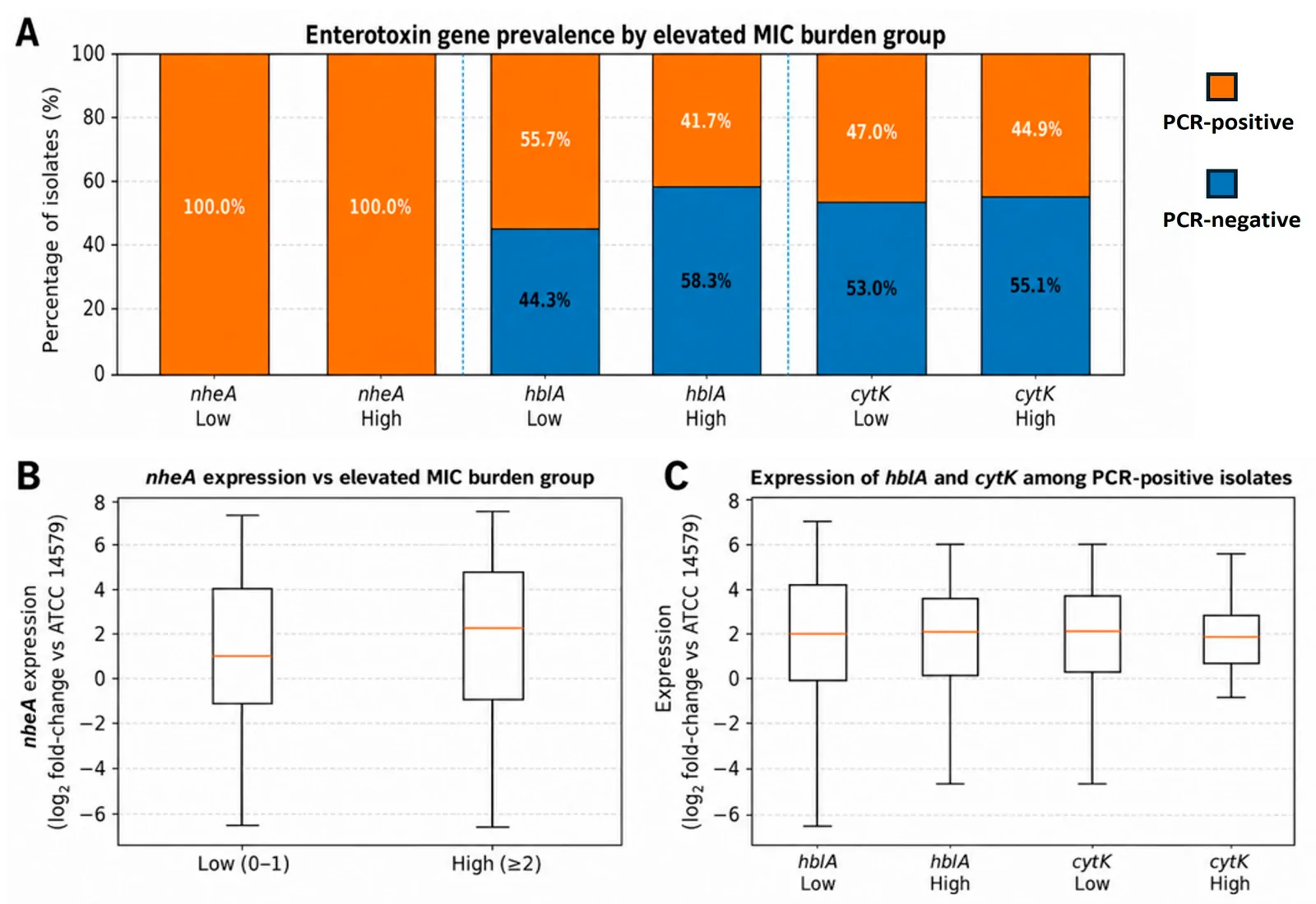
Enterotoxin gene prevalence and relative expression in relation to cumulative elevated MIC burden in *B. cereus* sensu lato isolates. (**A**) Prevalence of the enterotoxin-associated genes *nheA*, *hblA*, and *cytK* in isolates with low (0–1) or high (≥2) cumulative elevated MIC burden. Cumulative elevated MIC burden was defined as the number of antibiotics for which an isolate exhibited an MIC value exceeding the third quartile (Q3) of the overall distribution for that antibiotic. Orange segments represent PCR-positive isolates and blue segments represent PCR-negative isolates; percentages indicate the proportion of isolates within each burden group. (**B**) Relative expression of *nheA* in isolates with low and high cumulative elevated MIC burden. (**C**) Relative expression of *hblA* and *cytK* among PCR-positive isolates with low and high cumulative elevated MIC burden. Gene expression was calculated using the Pfaffl method and is presented as log_2_ fold-change relative to *B. cereus* ATCC 14579. A value of 0 therefore indicates expression equivalent to that of the reference strain, whereas positive and negative values indicate higher and lower expression, respectively. In panels (**B**,**C**), horizontal lines within boxes indicate medians, boxes represent the interquartile range (Q1–Q3), and whiskers extend to the most extreme values within 1.5 × IQR.

Analysis of enterotoxin gene distribution demonstrated that *nheA* was present in all investigated *B. cereus* s.l. isolates, irrespective of geographical origin or cumulative elevated MIC burden. The *hblA* and *cytK* genes were detected in approximately half of the strains. Comparison of gene frequencies between isolates with low (0–1) and high (≥2) elevated MIC burden did not reveal any differences for *nheA*. For *hblA* and *cytK*, only moderate fluctuations in prevalence were observed, without a clear trend associated with MIC burden level (Figure 5A).

Relative expression of enterotoxin genes, assessed using the Pfaffl method and expressed as log_2_-fold change relative to the reference strain *B. cereus* ATCC 14579, showed substantial variability among individual isolates but was not associated with elevated MIC burden. For *nheA*, no differences were observed between low- and high-burden groups (Mann–Whitney U test not significant; Spearman rank correlation analysis did not indicate a monotonic association). Similarly, among PCR-positive isolates for *hblA*, no differences in expression levels were detected between the analysed groups (U = 1676, *p* = 0.998), nor for *cytK* (U = 1521, *p* = 0.316) (Figure 5B,C).

Taken together, these results indicate that neither the presence nor the relative expression level of the analysed enterotoxin genes is significantly associated with cumulative elevated MIC burden in the studied population.

## 3. Discussion

To date, studies addressing antimicrobial resistance in opportunistic species such as *B. cereus* s.l. have been relatively scarce and have primarily focused on sporadic clinical infections, foodborne outbreaks, and food microbiology. An exception has been *B. anthracis*, owing to its pronounced pathogenicity in humans and animals. However, such a perspective has not permitted a comprehensive assessment of the role of anthropogenic pressure in shaping antimicrobial tolerance.

The present study demonstrates that the *B. cereus* s.l. population inhabiting soils adjacent to urbanised areas exhibits significant, albeit incomplete, geographical structuring. Reduced values of phylogeographic association indices (AI and PS) confirm that the distribution of phylogenetic groups is non-random, consistent with previous MLST-based analyses of the *B. cereus* s.l. complex, which have indicated the coexistence of widely distributed lineages and region-specific signals [21,22,23]. At the same time, the absence of clear continental segregation suggests that the population functions as a system of partially connected subpopulations, in which local diversification coexists with global dispersal.

In soil-dwelling bacteria, long-term spore persistence, environmental mobility of soil material, and anthropogenic transport of soil and agricultural products may enhance lineage flow at macrogeographic scales. Concurrently, processes such as purifying selection, previously documented in *B. cereus* s.l. [9,22], may further shape the genetic structure of the population.

Although latitudinal shifts in the distribution of selected clades may appear intuitive, the present findings support the occurrence of ecological filtering. The overrepresentation of psychrotolerant lineages at higher latitudes is consistent with earlier observations of adaptive ecological differentiation within the complex [22,24]. Adaptation to low temperatures involves modifications of membrane lipid composition, regulation of stress response pathways, reorganisation of central metabolism, and the synthesis of molecular chaperones stabilising macromolecular structures. Nevertheless, similarly to the findings of Tourasse et al. [21], the lack of strict regional segregation suggests that potential climatic filtering does not result in evolutionary isolation but rather coexists with global gene flow and additional processes enhancing genetic diversity.

Analysis of antimicrobial tolerance phenotypes revealed regional shifts in MIC distributions and variation in cumulative elevated MIC burden. Importantly, these patterns did not show a consistent association with local phylogenetic clustering, suggesting multiple and independent emergence of tolerance phenotypes. This may reflect convergent adaptation driven by exposure to similar environmental pressures across geographically distant regions. This interpretation is consistent with recent evidence indicating that urban soils may act as reservoirs of antimicrobial resistance, with local resistance patterns shaped by multiple anthropogenic inputs and environmental selective pressures [25].

Mechanistically, such patterns may involve mobile genetic elements, including plasmids, transposons, and integrative islands, as well as widely distributed chromosomal determinants, notably β-lactamases characteristic of the *B. cereus* s.l. complex. The importance of horizontal gene transfer in the environmental evolution of antimicrobial resistance has been extensively documented [26,27], as has the role of co-selection mediated by heavy metals and other anthropogenic stressors [28,29,30].

In this context, consideration of regional antibiotic consumption profiles is crucial. Global analyses of antimicrobial use indicate a persistently high proportion of broad-spectrum antibiotics classified within the WHO AWaRe framework as “Watch”, particularly in several Asian countries [31,32]. In China, β-lactams (including higher-generation cephalosporins), macrolides, and fluoroquinolones predominate, classes frequently assigned to the Watch category. This pattern translates into sustained and substantial selective pressure within both clinical and municipal environments [32,33]. The release of antibiotic residues via wastewater, sewage sludge, and surface runoff may maintain sublethal concentrations in the environment, thereby promoting the selection of tolerance phenotypes within soil microbiomes.

In Malaysia, the situation appears more complex. National data indicate that in the public healthcare sector, antibiotics from the Access group predominate; however, in the private sector, the proportion of Watch antibiotics is markedly higher [34]. Under tropical climatic conditions characterised by elevated temperature and humidity, increased microbial metabolic activity and intensified nutrient cycling may further modulate gene transfer dynamics and the stability of tolerance determinants. Consequently, soil environments may function as amplifiers of selective pressure originating within healthcare systems.

By contrast, in countries such as Germany and Canada, where extensive surveillance and antimicrobial stewardship programmes are implemented, antibiotic consumption patterns are more tightly controlled, with a relatively higher proportion of Access antibiotics and stricter monitoring of Watch-class agents [35,36]. This does not imply the absence of selective pressure, as broad-spectrum antibiotics remain integral to hospital-based therapy in these settings. However, the intensity and environmental dispersion of selective pressure may be mitigated through more effective wastewater management and tighter regulation of antibiotic use.

Taken together with the findings of the present study, these data suggest that regional differences in elevated MIC burden may result from a complex superposition of clinical, infrastructural, and environmental selective pressures. The broader tolerance ranges observed for multiple antibiotics in locations characterised by higher proportions of broad-spectrum antibiotic use are consistent with the hypothesis of environmental prolongation of selective pressure generated within healthcare systems. At the same time, the absence of a clear association with phylogenetic clustering indicates that this adaptation is multilayered and convergent, rather than confined to single expanding lineages.

The presence of *B. cereus* s.l. strains exhibiting reduced antimicrobial susceptibility and harbouring enterotoxin-associated determinants in urban and peri-urban soils may have implications extending beyond the soil ecosystem itself. Soil may serve as a reservoir of antimicrobial-resistant bacteria that can be dispersed with dust, soil particles, and plant material, creating opportunities for direct or indirect human exposure. This may be particularly relevant to urban and peri-urban agriculture, especially for crops consumed raw. Erosion and surface runoff may provide additional routes of dissemination by transporting soil-associated bacteria into surface waters and other connected environmental compartments. *B. cereus* s.l. has previously been shown to occur commonly in freshwater environments as both spores and vegetative cells [37], while strains harbouring enterotoxin genes and displaying antimicrobial resistance have also been detected in riverine and coastal environments [38]. These observations support consideration of environmental *B. cereus* s.l. populations within a broader One Health perspective. However, because the present study did not directly investigate resistance genes or transmission pathways between soil, plants, air, and water, these routes should be regarded as biologically plausible exposure scenarios rather than processes demonstrated experimentally in the present study.

The inclusion of gene expression analysis enabled quantitative assessment of the transcriptional activity of the investigated genes and provided an important complement to the genotypic data, as the mere presence of enterotoxin genes does not necessarily reflect their functional activity or the actual virulence potential of a given strain. The lack of a significant relationship between MIC burden and either the presence or expression level of enterotoxin genes supports the relative autonomy of evolutionary trajectories underlying antimicrobial tolerance and virulence potential. The ubiquity of the *nhe* operon and the variable distribution of *hbl* and *cytK* are consistent with previous observations of the widespread enterotoxigenic potential within environmental populations [39,40]. Regulation of these genes—mediated, among others, by the PlcR regulator and quorum-sensing systems—is conditional and multilayered, which may decouple selective pressures acting on environmental persistence from those shaping host interaction traits. Importantly, the accumulation of resistance traits observed here is unlikely to reflect inappropriate therapeutic use targeting foodborne infections, which are predominantly associated with toxigenic strains.

When interpreting these findings, it is essential to consider the intentional selection of isolates representing unique STs. This strategy enabled the analysis of phylogeographic patterns independently of local clonal dominance but limited inference regarding natural lineage frequencies and clonal expansion dynamics. Consequently, the observed relationships should be viewed as patterns within a representative yet structured dataset, rather than as a direct reflection of in situ population demography.

In summary, the observed geographic differences in antimicrobial susceptibility likely reflect the combined effects of local environmental selection and broader population-level processes. However, the present study does not directly identify the selective factors responsible for these patterns. Future analyses integrating antimicrobial resistance phenotypes with environmental measurements and whole-genome data will be necessary to determine the relative contributions of local selective pressures, horizontal gene transfer, and lineage-specific adaptation in shaping resistance within environmental *B. cereus* s.l. populations.

## 4. Materials and Methods

**Figure 6:**
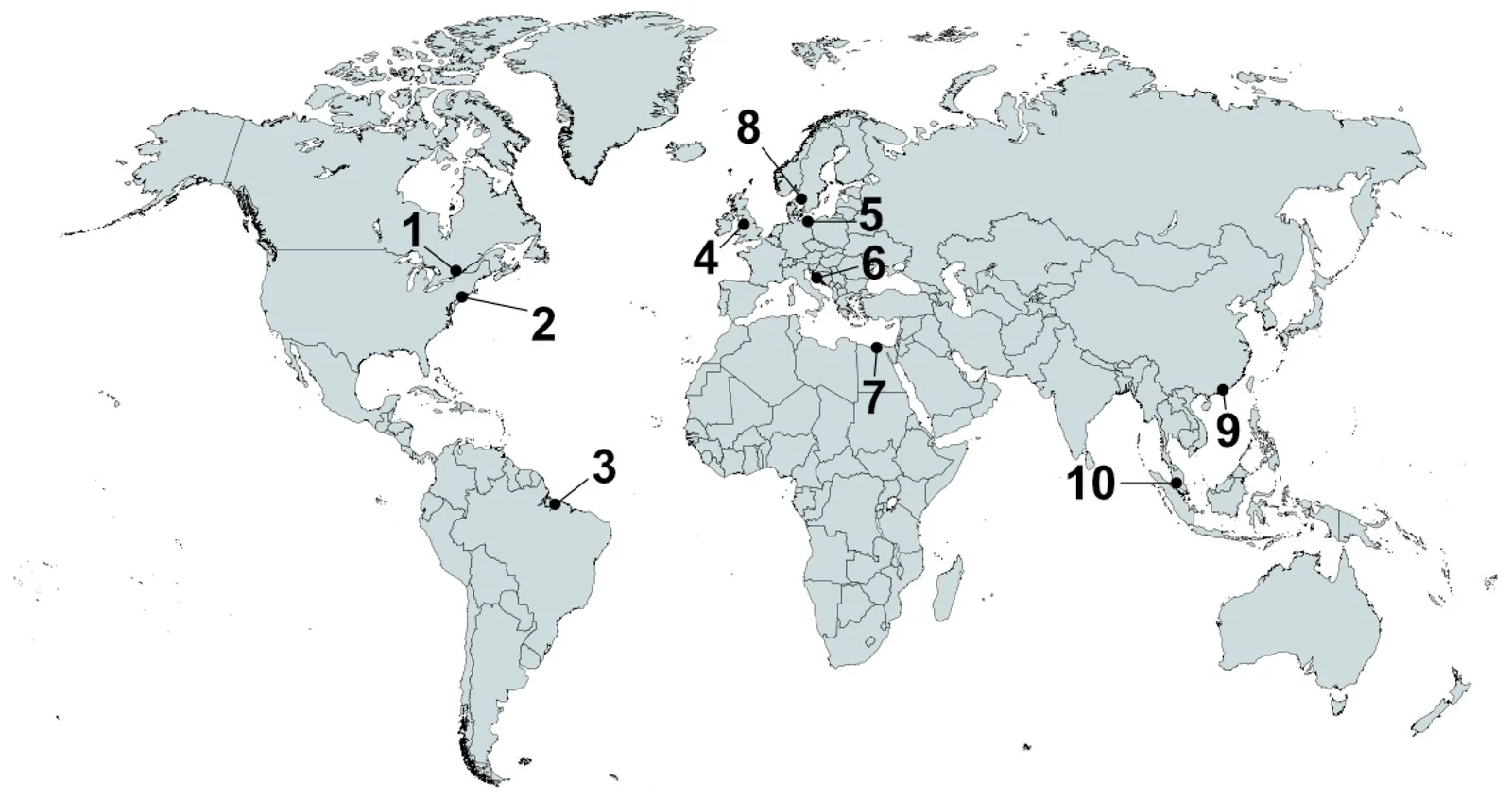
Sampling locations. Individual sampling sites: (1) Ottawa (Canada); (2) New York (USA); (3) Belem (Brazil); (4) Liverpool (United Kingdom); (5) Greifswald (Germany); (6) Zadar (Croatia); (7) Alexandria (Egypt); (8) Gothenburg (Sweden); (9) Shenzhen (China); and (10) Kuala Lumpur (Malaysia).

Soil samples were collected from ten locations situated within green areas of urban agglomerations across different continents: Ottawa (Canada), New York (USA), Belém (Brazil), Liverpool (United Kingdom), Greifswald (Germany), Zadar (Croatia), Alexandria (Egypt), Gothenburg (Sweden), Shenzhen (China), and Kuala Lumpur (Malaysia), as illustrated in Figure 6. All sampling sites were located within a maximum distance of approximately 10 km from city centres and were classified as green areas, including urban parks and flower meadows. The selection of these sites was intended to minimise the influence of direct point-source pollution, such as high-traffic roads, industrial facilities, or dense residential development, while capturing environments potentially characterised by long-term exposure to complex environmental factors associated with the functioning of large urban centres.

At each location, soil was aseptically collected from a depth of 5–10 cm using sterile tools and transferred into sterile containers. Samples were stored at room temperature and processed immediately upon transport to the laboratory. An initial pool of *B. cereus* s.l. isolates was obtained from each sampling location. Isolate selection for downstream analyses was subsequently based on MLST profiles, as described below. The final dataset comprised 243 *B. cereus* s.l. isolates. For comparative purposes, multilocus sequence typing (MLST) profiles retrieved from the https://pubmlst.org/bcereus (accessed on 10 August 2024) database were also included for *B. anthracis* Ames, *B. cereus* ATCC 14579, *B. mycoides* var. *weihenstephanensis* DSM 11821, and *B. cereus* F4810/72.

Isolation of bacteria from soil. Bacterial isolation was performed using microbiological methods commonly applied for the recovery of environmental spore-forming bacteria, in accordance with ISO 7932:2004 standards [41] for the enumeration of *B. cereus* in environmental products [42]. Soil samples were homogenised in sterile physiological saline (0.85% NaCl) using vortex mixers and mechanical homogenisers to ensure uniform dispersion of microorganisms. To selectively enrich for spore-forming bacteria, homogenates were subjected to heat treatment by incubation at 80 °C for 10 min, thereby reducing the abundance of non-spore-forming microorganisms. The resulting suspensions were subjected to serial decimal dilutions and plated onto selective and differential media, such as mannitol–egg yolk–polymyxin agar (MYP, Oxoid Ltd., Basingstoke, Hampshire, UK), which enables the detection of members of the *B. cereus* group. Bacteria were incubated under aerobic conditions at 30 ± 2 °C for 24–48 h. Large, dull, opaque colonies exhibiting lecithinase activity were selected for further analyses. Selected colonies were subcultured onto fresh growth media to obtain pure cultures, which were subsequently propagated and stored at −80 °C in cryoprotective medium for further analyses.

Phenotypic and molecular identification. Preliminary identification of isolates was based on classical morphological and phenotypic characteristics typical of *B. cereus* s.l., including Gram staining, haemolytic activity, and lecithinase production. Owing to the high degree of genetic and phenotypic similarity within the *B. cereus* s.l. group, isolates were collectively classified as *B. cereus* s.l., an approach widely accepted in population-based and environmental studies [43]. All isolates were additionally tested for their ability to grow at 7 °C and 43 °C. Adaptation to low temperature was defined as the capacity to proliferate at 7 °C accompanied by the absence of growth at 43 °C.

Nucleic acid extraction. Genomic DNA was extracted from 18-h bacterial cultures grown in Luria–Bertani (LB) broth (Oxoid Ltd.) at 30 °C following enzymatic cell lysis with lysozyme and subsequently purified using the DNeasy Blood & Tissue Kit (Qiagen, Hilden, Germany), according to the manufacturer’s protocol for Gram-positive bacteria, with the lysis step extended to 120 min to ensure optimal yield. DNA quality was assessed spectrophotometrically (at 230, 260, and 280 nm) using a NanoDrop 2000 instrument (Thermo Fisher Scientific, Waltham, MA, USA).

Total RNA was extracted from 18-h cultures grown in LB broth (Oxoid Ltd.) at 30 °C using the Total RNA Mini Plus Kit (A&A Biotechnology, Gdańsk, Poland), following the manufacturer’s instructions. The incubation temperature was selected to enable comparable growth of both mesophilic and psychrotolerant strains. RNA quality and integrity were verified spectrophotometrically and by agarose gel electrophoresis. Samples were treated with DNase (A&A Biotechnology) to eliminate residual genomic DNA. Complementary DNA (cDNA) synthesis was performed using the High Capacity cDNA Reverse Transcription Kit (Thermo Fisher Scientific) in a GeneAmp 9600 thermal cycler (Applied Biosystems, Foster City, CA, USA) under the following conditions: 10 min at 25 °C, 120 min at 37 °C, and a final inactivation step of 5 min at 85 °C.

Multilocus sequence typing and phylogenetic analysis. Genetic diversity and population structure were assessed using multilocus sequence typing (MLST). Internal fragments of seven housekeeping genes included in the established MLST scheme for the *B. cereus* group (https://pubmlst.org/bcereus (accessed on 10 August 2024)) were amplified by PCR and sequenced. The resulting sequences were compared with reference allele sequences available in the public *B. cereus* PubMLST database, allowing assignment of allele numbers and sequence types (STs). All allele sequences identified in the present study corresponded to alleles previously deposited and publicly available in PubMLST; therefore, no novel allele sequences requiring additional deposition in NCBI or another sequence repository were identified. Each assigned allele number corresponds to a defined nucleotide sequence in PubMLST, and the resulting ST uniquely specifies the respective allelic profile [9,42,43].

Following assignment of allele profiles and sequence types (STs), one representative isolate of each ST identified within a given sampling location was retained for downstream analyses. Thus, isolates sharing the same ST within the same location were excluded as local clonal replicates, whereas the same ST could be represented independently when recovered from different sampling locations. This approach was adopted to minimise the impact of locally overrepresented clonal lineages and to enable robust phylogenetic and phylogeographic comparisons across geographically distant regions.

Phylogenetic analysis was conducted using concatenated and aligned nucleotide sequences of the seven loci (*glpF*, *gmk*, *ilvD*, *pta*, *pur*, *pycA*, and *tpi*) corresponding to individual ST profiles. The best-fitting nucleotide substitution model was selected based on the lowest Bayesian Information Criterion (BIC) value. A phylogenetic tree was subsequently reconstructed using MEGA6 (Molecular Evolutionary Genetics Analysis version 6.0) [44]. Phylogenetic relationships were then integrated with antimicrobial susceptibility profiles and geographic origin data and visualised using Interactive Tree of Life (iTOL) v6 [45], enabling assessment of the association between phylogenetic structure and selected phenotypic and ecological parameters.

Antimicrobial susceptibility testing. Antimicrobial susceptibility was evaluated using both the disk diffusion method and determination of minimum inhibitory concentrations (MICs). Disk diffusion assays were performed using antibiotic-impregnated disks (Oxoid Ltd.) containing ampicillin, amoxicillin/clavulanic acid, clarithromycin, tetracycline, and gentamicin. The antimicrobial panel was selected based on previous susceptibility studies of *B. cereus* s.l., including those of Fiedler et al. [2] and Gao et al. [46], in which ampicillin and amoxicillin/clavulanic acid were evaluated as separate β-lactam susceptibility markers. Testing was performed according to CLSI disk diffusion procedures [47], and inhibition zone diameters were interpreted using CLSI-based criteria [48]. Because species-specific disk diffusion breakpoints are not available for *B. cereus* s.l., surrogate interpretative criteria were applied, consistent with approaches previously used for this bacterial group [46].

MIC values were determined using antimicrobial gradient strips (Liofilchem S.r.l., Roseto degli Abruzzi, Italy) for penicillin (PEN), amoxicillin/clavulanic acid (AMC), chloramphenicol (CHL), vancomycin (VAN), erythromycin (ERY), azithromycin (AZI), clindamycin (CLI), and rifampicin (RIF). Tests were performed on Mueller–Hinton agar (Oxoid Ltd.) under aerobic conditions, and MIC values were read and interpreted in accordance with the manufacturer’s instructions [9].

Detection of diarrhoeal enterotoxin determinants. The presence of *nheA* and *hblA*, representing the *nhe* and *hbl* enterotoxin operons, respectively, as well as the *cytK* gene encoding cytotoxin K, was determined by conventional PCR. Amplification reactions were performed using Hot Start PCR Mix (A&A Biotechnology) in a GeneAmp 9600 thermal cycler (Applied Biosystems). PCR products were separated by electrophoresis on 1–1.2% agarose gels, and the presence of the respective amplicons was used to determine the occurrence of the analysed enterotoxin-associated genetic determinants in the investigated isolates. Primer sequences used in this study are provided in Appendix A, Table A1.

Quantitative PCR. To assess the relative expression levels of selected genes associated with diarrhoeal enterotoxin production, quantitative real-time PCR (qPCR) analysis was performed. Complementary DNA (cDNA), synthesised from total RNA using the High-Capacity cDNA Reverse Transcription Kit (Applied Biosystems, Thermo Fisher Scientific), served as the template for amplification. qPCR reactions were performed in a final volume of 25 µL using RT PCR Mix SYBR (A&A Biotechnology) and gene-specific primers in a StepOnePlus Real-Time PCR System (Life Technologies, Carlsbad, CA, USA). All reactions were performed in triplicate. The annealing temperature was adjusted individually for each primer pair; the primer sequences and corresponding annealing temperatures are provided in Appendix A, Table A1.

Amplification efficiencies were determined separately for each primer pair using standard curves generated from five-point 10-fold serial dilution series. The slope of the linear regression obtained by plotting threshold cycle values against the logarithm of template concentration was used to calculate amplification efficiency. The specificity of amplification was assessed by melting-curve analysis. All reactions yielded a single distinct melting peak at the expected melting temperature, indicating the absence of detectable non-specific amplification products. No-template controls (NTCs) were included for each analysed gene.

The *udp* gene was used as the endogenous reference gene for normalisation of transcript levels. Relative gene expression was calculated according to the Pfaffl model [49], which accounts for differences in amplification efficiency between the target and reference genes. *B cereus* ATCC 14579 was used as the calibrator strain, and expression levels of the analysed genes were expressed relative to those detected in this strain.

Statistical and modelling analyses. Population structure and phylogenetic relationships within the *B. cereus* s.l. collection were inferred based on multilocus sequence typing (MLST) data. Phylogenetic reconstruction was performed using the Maximum Likelihood method under the GTR+G+I nucleotide substitution model, selected according to the Bayesian Information Criterion (BIC). Node support was evaluated using non-parametric bootstrap analysis with 1000 replicates. Reference strains representing the major phylogenetic lineages within the *B. cereus* s.l. group, as specified above, were included in comparative analyses.

To quantitatively assess the association between phylogeny and geographical origin, the Association Index (AI) and Parsimony Score (PS) statistics were calculated. These metrics quantify the extent of phylogenetic clustering of isolates with respect to a defined trait (sampling location). Statistical significance was evaluated using permutation tests in which location labels were randomly reassigned to tree tips to generate null distributions.

Local phylogenetic clustering within individual sampling sites was further assessed using the Monophyletic Clade (MC) statistic, which identifies clades enriched for isolates originating from the same geographical location. Statistical significance of MC values was determined by comparison with null distributions generated through random permutation of location labels. Because location-specific MC statistics were evaluated separately for ten sampling locations, nominal permutation *p*-values were additionally adjusted for multiple testing using the Benjamini–Hochberg false discovery rate (FDR) procedure.

Antimicrobial susceptibility data obtained by the disk diffusion method were analysed descriptively and comparatively across sampling locations. Given the limited availability of species-specific clinical breakpoints for the *B. cereus* s.l. group, susceptibility categories were interpreted using comparative criteria based on CLSI guidelines and published literature [45]. Associations between sampling location and the distribution of antimicrobial susceptibility phenotypes were assessed using chi-square tests.

Minimum inhibitory concentration (MIC) data were analysed to characterise the global landscape of antimicrobial susceptibility. For each antibiotic, MIC distributions were summarised using the median and interquartile range (IQR). To identify isolates exhibiting elevated MIC values independently of clinical breakpoints, a threshold based on the upper quartile (Q3) of the global MIC distribution was applied. Isolates with MIC values exceeding this threshold were classified as displaying elevated MICs. For each isolate, cumulative elevated MIC burden was calculated as the number of antibiotics for which the corresponding MIC exceeded the antibiotic-specific Q3 threshold.

Differences in relative enterotoxin gene expression between isolates with low and high cumulative elevated MIC burden were assessed using the Mann–Whitney U test. Associations between relative gene expression and cumulative elevated MIC burden were additionally evaluated using Spearman’s rank correlation.

Multivariate relationships among MIC profiles were explored using Principal Component Analysis (PCA). Prior to PCA, MIC values were log_10_-transformed. PCA was performed in R version 3.3.1 (R Foundation for Statistical Computing, Vienna, Austria) using the prcomp function. Variables were mean-centred according to the default settings of prcomp, without additional scaling to unit variance. PCA was used to reduce data dimensionality and to visualise similarities and differences in multidimensional MIC profiles among isolates and sampling locations.

Statistical analyses were performed using R version 3.3.1 (R Foundation for Statistical Computing, Vienna, Austria). Unless otherwise stated, statistical significance was defined as *p* < 0.05.

## 5. Conclusions

Urban-adjacent soils harbour genetically diverse *B. cereus* s.l. populations displaying substantial geographic variation in antimicrobial susceptibility and a widespread carriage of enterotoxin-associated determinants. Although isolates from the same locations showed significant phylogenetic clustering more often than expected by chance, antimicrobial resistance patterns were not consistently linked to local lineage structure, suggesting that elevated MIC phenotypes may arise across multiple genetic backgrounds. Geographic differences in antimicrobial susceptibility may reflect region-specific anthropogenic selective pressures, although this relationship requires direct environmental verification. Importantly, neither the presence nor the relative expression of *nheA*, *hblA*, and *cytK* was associated with cumulative elevated MIC burden. These findings support the view that urban-adjacent soils represent environmentally relevant reservoirs of *B. cereus* s.l. populations combining antimicrobial resistance and enterotoxin-associated potential, while these traits appear to follow largely independent evolutionary trajectories.

## Figures and Tables

**Table 1:** Location-specific phylogenetic clustering assessed using the Monophyletic Clade (MC) statistic. Nominal permutation *p*-values and Benjamini–Hochberg false discovery rate (FDR)-adjusted *p*-values are shown.

Sampling Location	Observed Maximum Monophyletic Clade Size (MC)	Nominal *p*-Value	FDR-Adjusted *p*-Value
Ottawa	2	0.5302	0.7932
New York	2	0.5552	0.7932
Belém	3	0.0289	0.1283
Liverpool	3	0.0385	0.1283
Greifswald	0	1.0000	1.000
Zadar	2	0.4808	0.7932
Alexandria	0	1.0000	1.0000
Gothenburg	0	1.0000	1.0000
Shenzhen	3	0.0285	0.1283
Kuala Lumpur	2	0.5212	0.7921

## Data Availability

The MLST allele sequences corresponding to the sequence types identified in this study are publicly available through the *Bacillus cereus* PubMLST database. Additional raw data supporting the conclusions of this article are available from the corresponding author upon reasonable request.

## Supplementary Materials

The following supporting information can be downloaded at https://www.mdpi.com/article/10.3390/ijms27188293/s1.

## Appendix A

**Table A1:** Nucleotide sequences and annealing temperatures of primers used for amplification of the enterotoxin-associated genes *nheA*, *hblA*, and *cytK*, encoding components of non-haemolytic enterotoxin (NHE), haemolysin BL (HBL), and cytotoxin K (CytK), respectively, as well as the housekeeping genes *glpF*, *gmk*, *ilvD*, *pta*, *pur*, *pycA*, and *tpi* used for multilocus sequence typing (MLST). Primer sequences for the MLST loci were obtained from the *Bacillus cereus* PubMLST database https://pubmlst.org/organisms/bacillus-cereus/primers (accessed on 10 August 2024).

Gene	Primer Sequence	Annealing Temperature (°C)	Product Size (bp)	Reference
*hblA*	F: AAGCAATGGAATACAATGGG R: AGAATCTAAATCATGCCACTGC	53	1154	[50]
*nheA*	F: TACGCTAAGGAGGGGCA R: GTTTTTATTGCTTCATCGGCT	56	499	[51]
*cytK*	F: GTAACTTTCATTGATGATCC R: GAATACTAAATAATTGTTTCC	50	505	[39]
*udp*	F: ACTAGAGAAACTTGGAAATGATCG R: GACGCTTAATTGCACGGAAC	58	101	[52]
*glpF*	F: GCG TTT GTG CTG GTG TAA GT R: CTG CAA TCG GAA GGA AGA AG	59	549	[53]
*gmk*	F: ATT TAA GTG AGG AAG GGT AGG R: GCA ATG TTC ACC AAC CAC AA	56	600	[53]
*ilvD*	F: CGG GGC AAA CAT TAA GAG AA R: GGT TCT GGT CGT TTC CAT TC	58	556	[53]
*pta*	F: GCA GAG CGT TTA GCA AAA GAA R: TGC AAT GCG AGT TGC TTC TA	56	576	[53]
*pur*	F: CTG CTG CGA AAA ATC ACA AA R: CTC ACG ATT CGC TGC AAT AA	56	536	[53]
*pycA*	F: GCG TTA GGT GGA AAC GAA AG R: CGC GTC CAA GTT TAT GGA AT	57	550	[53]
*tpi*	F: GCC CAG TAG CAC TTA GCG AC R: CCG AAA CCG TCA AGA ATG AT	58	553	[53]
